# Regulation of Three Virulence Strategies of *Mycobacterium tuberculosis*: A Success Story

**DOI:** 10.3390/ijms19020347

**Published:** 2018-01-24

**Authors:** Niels A. Zondervan, Jesse C. J. van Dam, Peter J. Schaap, Vitor A. P. Martins dos Santos, Maria Suarez-Diez

**Affiliations:** 1 Laboratory of Systems and Synthetic Biology, Wageningen University & Research, Stippeneng 4, 6708 WE Wageningen, The Netherlands; nazondervan@gmail.com (N.A.Z.); jesse.vandam@wur.nl (J.C.J.v.D.); peter.schaap@wur.nl (P.J.S.); vitor.martinsdossantos@wur.nl (V.A.P.M.d.S.); 2 LifeGlimmer GmbH, Markelstrasse 38, 12163 Berlin, Germany

**Keywords:** *Mycobacteria*, virulence, immune modulation, dormancy, escape, phagosome rupture, divalent metal, pore, cAMP, manganese, iron, zinc, esx

## Abstract

Tuberculosis remains one of the deadliest diseases. Emergence of drug-resistant and multidrug-resistant *M. tuberculosis* strains makes treating tuberculosis increasingly challenging. In order to develop novel intervention strategies, detailed understanding of the molecular mechanisms behind the success of this pathogen is required. Here, we review recent literature to provide a systems level overview of the molecular and cellular components involved in divalent metal homeostasis and their role in regulating the three main virulence strategies of *M. tuberculosis*: immune modulation, dormancy and phagosomal rupture. We provide a visual and modular overview of these components and their regulation. Our analysis identified a single regulatory cascade for these three virulence strategies that respond to limited availability of divalent metals in the phagosome.

## 1. Introduction

*Mycobacterium tuberculosis* (*Mtb*) is the most successful known intracellular pathogen infecting roughly one third of the world population and killing about 1.3 million people in 2017 alone [1]. Treating *Mtb* infection is increasingly difficult due to increasing number of drug-resistant, multidrug-resistant and extensively drug-resistant strains [1]. In order to come up with new drug targets and treatment strategies, there is an urgent need to understand the molecular mechanisms supporting the success of this versatile pathogen. Here, we will review the regulation of three important survival strategies of *Mtb*: immune modulation, dormancy and phagosomal rupture [2,3,4].

Firstly, *Mtb* is a master in immune modulation. Its ability to interfere with host cell signalling pathways allows it to carefully balance production of cytokines involved in activation of the pro-inflammatory and anti-inflammatory response [5,6]. By balancing the pro- and anti-inflammatory immune response, *Mtb* delays phagosome maturation, harvests essential nutrients and stimulates the formation of granulomas. At early infection states, these granulomas are initially dominated by alveolar macrophages and shield the bacteria from more effective immune cells [7].

Secondly, when residing in the hypoxic granuloma, *Mtb* enters a metabolically near inactive and non-replicating dormant state in which it is immune to most types of drugs [8]. *Mtb* manipulates the macrophages to accumulate lipids, providing it with the nutrients required to sustain dormancy for multiple decades [7,9,10,11,12].

Thirdly, *Mtb* has a highly regulated pore formation system that it uses to rupture the phagosome and gain cytosolic access, resulting into necrosis of the host cell and dissemination of the bacilli [13,14].

The fine-tuned regulation of these three virulence strategies is what makes *Mtb* such a successful pathogen. A large body of literature exists on these virulence strategies and on their molecular components. However, there have been few attempts to provide a systems wide overview of these three virulence strategies, their molecular components and their regulation. Divalent metals play an important role in the regulation of some key aspects of these strategies [15,16,17]. Here, we will present an overview of their involvement in this regulatory process. Detailed inspection of available knowledge pinpoints a single regulatory cascade as a main control hub for these three virulence strategies, representing their interconnectivity as subsequent stages encountered in pathogen host interaction. A modular overview of the molecular components involved in divalent metal homeostasis and their components involved in these three virulence strategies can be found in Figure S1 and Table S1. In the following, we will discuss these components and the environmental cues that control them and we will highlight the role of divalent metals in the phagosome.

## 2. Divalent Metals at the Interface of *M. tuberculosis* Host Interaction

Divalent metals such as iron, zinc and manganese are required for proliferation and survival of all living organisms. Divalent metals appear, in all living beings, nearly exclusively as constituents of proteins and act as cofactors in many essential enzymes and environmental sensors [18]. Iron is the most commonly used divalent metal cofactor [18]. Iron containing enzymes are involved, among other processes, in electron transfer, maintaining redox balance and detoxification [19]. Manganese has the strongest affinity for ATP and is the preferred cofactor in cAMP production [20,21]. Zinc is used as cofactor by numerous enzymes and DNA binding proteins and additionally functions to scaffold additional proteins [22].

To prevent growth of bacteria, the host uses high affinity iron binding proteins such as lactoferrin, ferritin and transferrin to keep concentration of free iron in the blood low, in the so-called iron sparing response [16,23]. These proteins also bind other divalent metals such as manganese, albeit with lower specificity than iron. Similarly, calprotectin functions as high affinity calcium binding protein but also binds manganese, zinc and iron in the blood [24]. During infection, macrophages withdraw approximately 30% of the total circulating iron from the blood stream making macrophages environments rich in divalent metals [25]. Some intracellular pathogens use this defence mechanism to their advantage by stimulating phagocytosis by macrophages to get access to divalent metals and other nutrients. During initial infection, *Mtb* predominantly encounters resident, replicative alveolar macrophages populating the lungs which are rich in divalent metals while having reduced bactericidal abilities compared to other macrophages [12,25].

Upon ingestion by a macrophage, *Mtb* is engulfed in a special compartment called the phagosome, in a process known as phagocytosis. The phagosome then fuses with vesicles containing enzymes and other proteins that facilitate bacterial digestion. Phagocytosis is a rapid process and leads to phagosomal-endosomal fusion in approximately 3–4 min, acidification of the phagosome within 23–32 min and fusion with lysosome in 74–120 min, based on experiments with epithelial macrophages [26]. However, *Mtb* blocks phagosome maturation in an early phase leading to fusion with early endosomes and a pH of approximately 5.5 [27].

The macrophage continuously exports divalent metals out of the phagosome via Nramp1 and Nramp2 in a pH dependent manner. Many cell types express Nramp2 while only macrophages express Nramp1. Nramp1 is mechanistically similar to Nramp2 but has a much higher specificity for manganese (Mn) compared to Nramp2 [17,27,28]. Mn is required as cofactor for the bacteria to break down oxidative compounds produced in the phagosome such as H_2_O_2_ [16,20,29]. Thus, restricting Mn availability in the phagosome by recruitment of Nramp1 is an essential defence against intracellular pathogens. Nramp2 functions optimally around pH 6, a condition found in the early phagosome while Nramp1 has an optimal activity at a pH of 4.5 Nramp1 is attached to the membrane of maturing phagosomes and is associated with increased recruitment of endosomes and/or lysosomes containing vacuolar V-H^+^-ATPase, resulting in acidification of the phagosome from pH 6.5 to 5.5 [27,30]. Nramp2 is regulated separately from Nramp1 and co-localizes with transferrin receptors to early endosomes as well as with V-H^+^-ATPase. V-H^+^-ATPase provides the electro-genic force needed for Nramp1 and Nramp2 to operate [31,32]. Metal availability in the phagosome is tightly regulated by the host through the combined action of Nramp1 and Nramp2. Therefore, blocking phagosome maturation is an effective strategy to create an environment in which *Mtb* can outcompete divalent metal export from the phagosome. *Mtb* uses special high affinity siderophores (mycobactin) to gain access to divalent metals from both extracellular transferrin and the intracellular iron pool [25].

**Figure 1:**
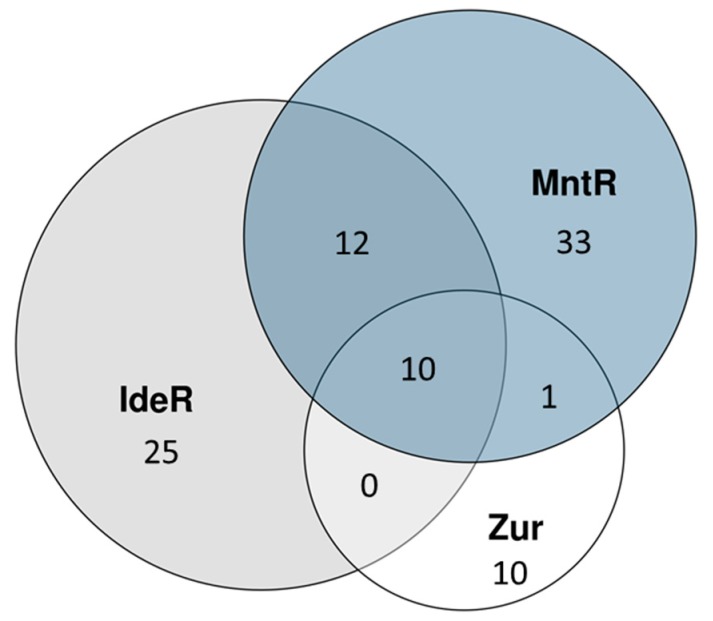
Number of genes in the IdeR, Zur and MntR regulons.

Within *Mtb* iron, zinc and manganese homeostasis are regulated by IdeR, Zur (previously known as FurB) and MntR respectively [19,22,33]. Ligation of Fe^2+^ to IdeR and Zn^2+^ to Zur stabilizes the formation of dimers that have strong affinity to binding sites involved in suppressing the genes in their respective regulons [15,19,34]. MntR in *Bacillus subtilis* contains two manganese binding sites as well as a dimerization site similar to IdeR and Zur [35]. There is a significant overlap between IdeR, Zur and MntR regulated genes, see Figure 1. An overview of the regulation of molecular components by divalent metal regulators, IdeR, Zur and MntR can be found in Figure S1 and Table S1. Each of these three regulators suppresses the main operon of genes coding for the ESX-3 secretion system and associated PE, PPE and Esx proteins homologues of ESAT-6 and CFP-10 (EsxA and EsxB) [33]. We will further discuss the ESX-3 transport system in a section below. In the following sections, we will discuss main characteristics of genes regulated by Fe, Zn and Mn respectively.

### 2.1. Iron Homeostasis and Redox Sensing

*Mtb* produces high affinity hydrophilic and lipophilic siderophores termed carboxy-mycobactin and mycobactin, respectively. Mycobactin can bypass the phagosome membrane to scavenge iron from the extracellular iron storage protein transferrin [25,36,37,38]. In addition, *Mtb* actively synthesizes deoxy-mycobactin during iron starvation [39].

*Mtb* combines the expression of a dedicated iron acquisition machinery with cellular components involved in immune modulation. By limiting acidification of the phagosome, *Mtb* maintains favourable conditions in which it can outperform active export of divalent metals by the macrophages transporter Nramp1. *Mtb’s* success in acquiring iron is illustrated by a 20-fold increase of iron concentrations in the phagosome between 1 and 24 h of macrophage infection [40]. However, high iron concentrations renders *Mtb* much more vulnerable to the formation of oxygen and nitrogen radicals upon phagosome maturation, as iron functions as a catalyst in the formation of radicals via the Fenton reaction [41]. Tight regulation of iron homeostasis is, therefore, essential, making IdeR an interesting drug target [42]. *Mtb* has adapted to deal with oxidative stress outside of the cell but is relatively vulnerable to endogenously generated oxidative stress in comparison to *M. smegmatis* [41]. Due to this vulnerability, vitamin-C is an effective drug to combat *Mtb* in the early stage of infection by inducing the Fenton reaction in iron rich phagosomes [43]. The oxidative conditions encountered in the phagosome leads to oxidation of the intracellular iron pool. Oxidation of the iron pool de-represses IdeR regulated genes among which some are involved in virulence. Upregulating expression of virulence genes in low iron and oxidative conditions is a common response in intracellular pathogens and has been observed in *Shigella dysenteriae*, *Corynebacterium diphtheniae*, *Yersinia pestis* and *Yersinia pseudotuberculosis*, as well as in *Mtb* [44,45].

The iron pool within *Mtb* and the phagosome functions as redox sensor to the oxidative conditions encountered in the early phagosome. In oxidative conditions, ferrous iron (Fe^2+^) is oxidized to ferric iron (Fe^3+^) [46]. Ferric iron does not bind to IdeR, leading to upregulation of IdeR suppressed genes in oxidative conditions [42]. Genes suppressed by IdeR code for proteins involved in siderophore synthesis (*mbtA-G*), secretion (*mmpL4/5*, *mmpS4/5*) and uptake (*irtAB*) as well as 11 genes coding for the ESX-3 secretion system, among others [47,48,49]. Even though IdeR mainly functions as iron dependent repressor, IdeR also induces transcription of four genes. Among the induced genes, *bfrB* and, to a lesser extent *bfrA*, code for mycobacterial ferritin-like iron storage proteins, which prevent overload of iron within *Mtb* [19,50]. Analysis of the promoter region of *bfrB* revealed it contains two tandem IdeR binding sites involved in alleviating repression by Lsr2. Lsr2 is a histone like regulator that binds AT-rich regions virulence islands, including those coding for ESX-1, espACD and PDIM coding genes, acting as a global regulator to aid in the adaptation to extremes in oxygen availability [50,51,52,53,54,55]. Combined regulation of *bfrB* by Lsr2 and IdeR, suggests iron storage by BfrB is suppressed by Lsr2 during infection under changing oxygen conditions unless IdeR detects availability of intracellular ferrous iron which indicates a lack of oxidative conditions. Under low iron conditions, BfrA is required to mobilize stored iron. On the other hand, on high iron conditions, BfrB is needed for iron storage [56]. BrfB was shown to be required for the long term persistence of *Mtb* in iron-starved granulomas [23].

Iron homeostasis is an essential process for bacterial survival, therefore its cellular components are interesting drug targets. This was shown in a knockout study of the *mmpS4/5* siderophore secretion, which resulted in limited intracellular availability of iron as well as intracellular accumulation of siderophores toxic to *Mtb* [57]. Another interesting drug target is HupB, a nucleoid-associated protein that protects *Mtb* against reactive oxygen species, regulates siderophore synthesis and was proposed to facilitate transfer of iron from ferri-carboxymycobactin to mycobactin [58,59]. HupB stimulates transcription of its own operon in the absence of IdeR-Fe^2+^ [59].

IdeR also regulates genes involved in response to oxidative and acidic stress, among which the two-component system PhoPR. Two-component systems contain a histidine kinase sensor that senses specific environmental stimulus and a response regulator that gets phosphorylated by the sensor upon specific environmental stimuli. Many two-component regulators, among which PhoPR, also regulate their own operon [60]. Presence of multiple binding sites allows both positive and negative regulation depending on the concentration and phosphorylation state of the response regulator, as is the case for PhoPR [61,62]. PhoPR is the main regulator of the oxidative and acidic stress response but also it is the initial step in a regulatory cascade controlling pore formation and phagosomal rupture. Six putative IdeR binding sites upstream of the *phoP-phoR* operon were located, of which five were observed to bind IdeR in the presence of iron [63]. This points to a possible link between iron homeostasis and PhoPR regulation of the oxidative stress response and virulence genes.

Nevertheless, the exact role of IdeR in upstream binding of PhoPR remains to be determined.

Oxidation of the iron pool is also sensed by proteins containing iron-sulphur clusters such as the enzyme aconitase (Acn) and the regulators FurA and WhiB1-7. Acn catalyses the isomerization of citrate to isocitrate via cis-aconitate in normal conditions. However, in low iron or oxidative conditions it binds to and suppresses translation of IdeR-mRNA while increasing translation of TrxC-mRNA [64]. The function of Acn as redox sensitive translational regulator is conserved in many organisms [46,65].

FurA (ferric uptake regulator A) regulates the oxidative stress response by modulating expression of the operon coding for FurA and the KatG catalase [66]. KatG is essential for the breakdown of H_2_O_2_ radicals formed upon phagosome endosome fusion and activates the anti-cell-wall drug isoniazid. Recently, transcriptional activation of *furA-katG* was found to be regulated by RbpA, which is induced by H_2_O_2_ in a SigE dependent manner [67].

A third iron sensitive regulator is WhiB7. WhiB proteins are iron-sulphur cluster-containing redox-sensing transcription factors. WhiB7 expression is auto-regulated by binding to its own promoter in response to antibiotics or redox stress [68]. An 80-fold upregulation of WhiB7 was observed upon treatment with antibiotics that bind to the 30S ribosomal subunit such as kanamycin and streptomycin [68]. WhiB7 is upregulated by iron starvation and was shown to induce transcription of *eis* and *tap* [69], two antibiotic resistance genes. Upregulation of *eis* increases secretion of IL-10 and slightly represses production of TNF-α by the host. IL-10 and TNF-α are involved in the anti-inflammatory and pro-inflammatory responses respectively [70].

In summary, oxidation of the iron pool is an important environmental cue to activate molecular components involved in iron sequestering, immune modulation and virulence. IdeR, FurA, Acn, WhiB7, Lsr2 and SigE are all involved in the response to the oxidative conditions encountered in the phagosome and subsequent adaption through expression of a vast repertoire of molecules involved in iron homeostasis as well as genes involved in modulation of the immune response.

### 2.2. Manganese Homeostasis and cAMP Production

Manganese is one of the most abundant metal elements in nature [71]. Mn is involved in enzymes of diverse functionality such as photosynthesis and detoxification: Mn is used as cofactor for both synthesis and degradation of H_2_O_2_, superoxide and radicals [16]. The oxidative burst is a very effective bactericidal process to defend against intracellular pathogens such as *Mtb* and *Y. Pestis* [54,72,73]. As previously stated MntR is a regulator of Mn homoeostasis, however MntR is dispensable for *Mtb* growth in human and/or mice macrophages due to the limited availability of Mn in the phagosome. Manganese transport on the other hand is required for virulence and to break down oxygen radicals [33]. *Mtb* contains two superoxide dismutases, SodA and SodC. SodA uses manganese as preferred cofactor and requires CtpC for metalation and export to the phagosome. Interestingly, *ctpC* transcription is induced in the presence of PhoP, while *sodA* is predicted to contain upstream cAMP-CRP binding sites implicating it in its regulation [60,74]. CRP is a cAMP dependent regulatory protein.

Another role of Mn we would like to discuss here is the Mn dependent activation of cAMP production in the early phagosome which was first proposed by S. Reddy et al. in 2001 [21]. S. Reddy and co-workers studied the kinetics of membranes containing *Mtb* adenylyl cyclase CyA (Rv1625c). Their study revealed that the Michaelis-Menten constant (Km) for Mn-ATP is 70-fold lower than for Mg-ATP. This results in a 47-fold activation by 1 mM Mn-ATP compared to 1 mM of Mg-ATP at physiological conditions [21]. Mn is also essential for the CRP regulated, virulence associated type III phosphodiesterase Rv0805 [75,76].

During infection, intracellular cAMP concentration increases ~50 fold and this is associated with a decrease in pH from 6.7 to 5.5 [77]. Among the 15 Adenylate Cyclases (AC) present in *Mtb* H37Rv, CyA has the highest measured cAMP production while AC (Rv1264) functions optimally at pH 6, which is typically found in early phagosomes [77,78]. *Mtb* was shown to secrete cAMP in a burst into the macrophage cytosol, resulting in a 10-fold increase in the host’s TNF-α concentration, an important inducer of granuloma formation [79]. Rv0386 is needed for this cAMP burst [79].

The MntR regulon contains *mntH* (Rv0924c), coding for Mramp, an Nramp homolog that imports manganese (Mn) in a pH dependent manner; *mntABCD* (Rv1283c-Rv1280c) coding for an ATP dependent manganese transporter and *Rv2477c* coding for a manganese dependent ATPase which optimally functions at pH 5.2 [80]. Interestingly, Rv2477c was postulated to be involved in resistance to tetracyclines and macrolides [80]. Additionally, MntR and Zur regulate *Rv2059-Rv2060* coding for two components of an incomplete ABC transporter of unknown function. Therefore, it is more likely that this transporter is involved in transporting other divalent cations like Co^2+^, Cu^2+^ or Ca^2+^ to substitute Mn and Zn in some conditions. A second possibility is that this operon codes for a divalent cation exporter to counter the side effect of unwanted uptake of divalent cations such as Cu^2+^ by the high expression of manganese and zinc transporters [33]. Manganese uptake plays an important role in virulence of many bacteria. For instance, supplementing *Salmonella typhimurium* with manganese prior to infecting macrophages, decreased its lethal dose 50-fold [81]. Similarly, manganese acquisition in the gut was shown to allow *S. typhimurium* and *Salmonella enterica* to evade neutrophil killing by calprotectin and reactive oxygen species, while patients with mutations in manganese transporter Nramp1 were shown to be much more susceptible to pathogens such as *Mtb* [20,27,54,72,82,83]. 

MntR regulates WhiB6 which regulates *espACD* and some DevR (previously known as DosR) regulated genes [84]. DevR is the main regulator of dormancy and *espACD* is involved in pore formation [85] and will be discussed below. The WhiB6 iron sulphur cluster is necessary for the negative control of the DevR regulon and positive control of the ESX-1 secretion system, whereas apo-WhiB6 induces the DevR regulon and suppresses ESX-1 expression in *M. marinum* [85]. A model was proposed where holo-WhiB6 positively regulate ESX-1 operon while upon reaction with reactive oxygen species and NO, apo-WhiB6 and WhiB6-DNIC are formed respectively. Both apo-WhiB6 and WhiB6-DNIC activate DevR regulated genes to shift metabolism and maintain energy and redox homeostasis [85].

MntR interacts with the toxin-antitoxin system RelJ and RelK in which MntR functions as antitoxin [86,87]. Additionally, VapBC26 and VapB30 toxin-antitoxin system both requires Mg or Mn for their ribonuclease activity, which inhibits growth [88,89]. These results indicate Mn might function as environmental cue in the regulation of growth.

### 2.3. Zinc Homeostasis

The third and final divalent cation we would like to discuss is zinc, the only redox stable divalent metal of the three. As previously stated, zinc homeostasis is regulated by Zur (FurB), a Zn^2+^ dependent repressor. Zur knockout studies identified 32 genes that are upregulated in the *zur* knockout mutant of which 24 belong to eight transcriptional units that were shown to be directly regulated by Zur [22]. Zur expression levels are regulated by SmtB encoded by an upstream gene, which is co-operonic with *zur*. SmtB functions as a repressor which is deactivated upon binding to Zn^2+^ [22].

There are three possible zinc uptake systems regulated by Zur. Firstly, Zur regulates the *sitABC* like genes (*Rv2059-2060*), which are also regulated by MntR that were previously discussed. This suggest that this transporter might function as Zn importer [20,90,91]. Secondly, Zur regulates *Rv0106* coding for a protein similar to the *B. subtilis* putative zinc low-affinity transporter YciCas [90]. Thirdly, EsxG-EsxH proteins were shown to be able to bind zinc, which might implicate them in zinc transport [92].

Other interesting targets of Zur are five genes coding for ribosomal proteins that can function in the absence of zinc, in contrast to their zinc dependent counterparts which normally bind to the 30S ribosomal subunits [22,93]. Although Zur was found to be able to positively regulate some genes in other pathogenic bacteria via repression of non-coding small RNAs, no such regulation was found in a *zur* knockout *Mtb* mutant [15].

### 2.4. ESX-3 Secretion System

The ESX-3 secretion system is the only one of the five ESX systems that is essential for in vitro growth of Mtb [94,95]. ESX-3 is involved in divalent metal homeostasis and immune modulation. ESX-3 is involved in divalent metal homeostasis and immune modulation. ESX systems secret extracellular proteins [96,97].

Regulatory binding site for all three divalent metal regulators IdeR, Zur and MntR can be found in the ESX-3 core operon promoter [48,92], as summarized in Table 1. The triple control of ESX-3 might allow *Mtb* to switch partly to other divalent metals in the absence of one of these three. This hypothesis is supported by the observation that siderophore knockout mutants low in iron contain much higher zinc concentrations [32]. However, many ESX-3 associated genes are regulated by only one or two of these regulators, indicating dedicated roles in homeostasis of specific metals [98].

All three divalent metal regulators regulate EsxG and EsxH which play an essential role in secretion of PE and PPE proteins [98]. PE and PPE proteins comprise nearly 10% of the coding potential of the *Mtb* genome and, for many of them, immune modulating properties have been reported [100]. A large number of studies exist on the immune modulating properties of ESX-3 secreted PE and PPE proteins [95,98,100,101,102,103,104,105]. The ESX-3 secreted protein pair EsxG-EsxH, targets the endosomal sorting complex to impair fusion of the phagosome with the lysosomes, while increasing association with the endocytic pathway leading to fusion with transferrin containing vesicles [92,95,97]. PE5-PPE4 were found to be critical for the siderophore-mediated iron-acquisition functions of ESX-3 [98]. PPE38 inhibits macrophage MHC Class I expression, dampens CD8+ T-Cell responses and was shown to be required for virulence of *M. marinum* [104,105]. PPE37 was found to reduce the production of pro-inflammatory factors TNF-α and IL-6 [102]. PE_PGRS61 binds TLR2 in a Ca^2+^ dependent manner, leading to increased IL-10 production. Finally, PE5 and PE15 trigger activation of the host MAP kinases required for IL-10 production [100,103]. IL-10 is an important anti-inflammatory cytokine. IL-10 reduces the expression of *iNOS*, limiting production of nitric oxide (NO) in the phagosome [95,100]. Enhanced IL-10 expression plays an important role in inhibiting early protective immunity and blocking phagosome activation [106,107]. In addition, a direct role for IL-10 in *Mtb* reactivation has been observed [106]. Interestingly, IL-10 also modulates lipid metabolism by enhancing uptake and efflux of cholesterol in macrophages [106,107,108]. *Mtb* is known to induce foamy macrophages using immune modulating proteins as well as secreted lipids. This leads to deregulation of the macrophages lipid metabolism via the macrophages’ lipid-sensing nuclear receptors PPARγ and TR4 [12,107]. One study reported observing *Mtb* to exploit host vesicle trafficking and lipid storage by recruitment of iron bound mycobactin to lipid droplets which move to the phagosome and discharge their content [36]. Another study found that *Mtb* uses membrane vesicles containing immune modulating molecules as well as mycobactin to interact with the macrophage during infection [109]. Further research is needed to investigate the proposed synergy between modulation of host vesicle trafficking, lipid acquisition and iron acquisition.

## 3. Three Main Virulence Strategies of *Mtb*

The three virulence strategies discussed in this review, namely immune modulation, dormancy and phagosomal rupture, represent subsequent stages in *Mtb*-host interaction. These strategies extend and complement each other, which is reflected in their regulation. While many pathogens directly express components involved in phagosomal rupture, *Mtb* keeps a low profile and activates key virulence strategies, such as phagosomal rupture, only when immune modulation fails and the phagosome becomes inhospitable. However, immune modulation also complements phagosomal rupture and dormancy, since immune modulation leads to conditions, such as granuloma formation and cholesterol accumulation, which are needed to prepare *Mtb* for dormancy and phagosomal rupture.

### 3.1. Immune Modulation

*Mtb* uses a number of virulence proteins, complex lipids and secreted metabolites, to modulate the immune response and arrest phagosome maturation to prevent fusion with late endosomes and lysosomes [2,77,97,110,111,112,113]. In case of successful immune modulation, phagosome maturation is halted resulting in a pH of approximately 5.5 [27,30]. The macrophage controls intracellular trafficking, including phagosome maturation, through 42 distinct Rab GTPases. Rab5 is associated with phagosomes immediately after phagocytosis and normally diffuses quickly, allowing Rab7 to associate to the phagosome, which allows fusion of the phagosome with lysosomes. Studies with *M. bovis* have shown that *Mycobacteria* halts phagosome maturation, by blocking vesicle fusion between stages controlled by Rab5 and Rab7, with no Rab7 being accumulated in macrophages even after 7 days [111]. Similarly, for *Mtb* Rab7 was shown to be recruited by the phagosome but its premature release prevents fusion of the phagosome with late endosomes [110,114].

In addition to the earlier discussed ESX-3 secreted proteins, several other proteins and molecules are involved in blocking phagosome maturation. Secreted tyrosine phosphatase (PtpA) is involved in the exclusion of the vacuolar V-ATPase, thereby preventing acidification and fusion with lysosomes [112,115]. cAMP secreted by *Mtb* blocks phagosome lysosome fusion by inhibiting actin assembly [113]. Additionally, a number of virulence lipids interfere with the phagosome’s Golgi trafficking, needed for maturation of the phagosome [114,116]. Among these virulence lipids are monomycolate, dimycolate, sulpholipid-1, diacyl trehalose, polyacyl trehalose as well as phthiocerol dimycocerosate (PDIM). Of these lipids, PDIM was shown to play a role in phagosomal rupture and will be discussed in the section below.

**Figure 2:**
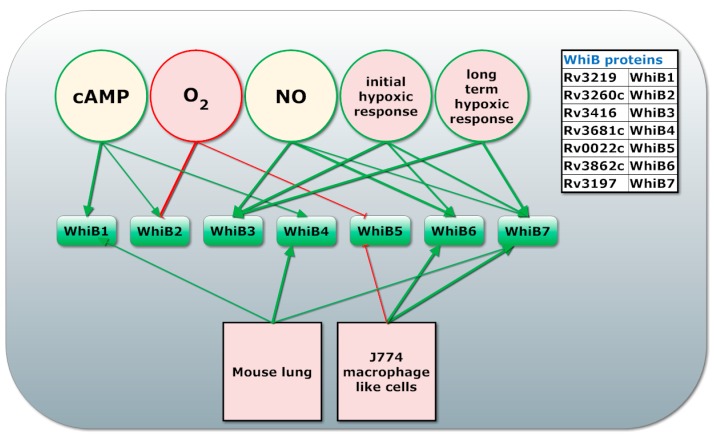
WhiB1-7 transcriptional response to environmental stresses. Proteins from the WhiB family are presented in the squares. The circles in the top indicate environmental cues (O_2_, NO, cAMP availability) or infection stages (initial or long term hypoxic response). Squares represent different environments (mouse lung and JJ774 macrophage like cells). Arrows indicated regulation (green for induction, red for inhibition of transcription) with the line width indicating the strength of the interaction based on the fold change of their transcript level in a given conditions [68,118].

*Mtb* is very successful in balancing the expression of molecular systems involved in activating the pro- and anti- inflammatory responses of the host to direct the immune response to favourable conditions for its survival. *Mtb* achieves this balance through multitude sensors and that integrate many environmental cues. One important family of regulators involved in sensing internal conditions are the iron-sulphur cluster containing WhiB family of regulators, already mentioned in the section on iron homeostasis. Different WhiB regulators have different redox potential and sensitivity to oxidative agents such as O_2_ and NO and for some, thioredoxin like protein disulphide reductase activity has been reported [68,117,118,119]. Many *whiB* genes are regulated by cAMP-CRP [68], as summarized in Figure 2.

WhiB1 is an essential regulator that senses NO, is regulated by cAMP-CRP and is associated with resuscitation [119,120]. WhiB4 is associated to the oxidative stress response while WhiB5 is required for resuscitation [121,122]. DNA binding has only been experimentally proven for WhiB1, WhiB2, WhiB3, WhiB6 and WhiB7 [68,85]. Interestingly, WhiB1-3 are induced during infection and, upon nutrient limitation, by exogenous cAMP. This indicates they are involved in sensing the redox state of *Mtb* [123]. For WhiB1-3 it was shown that their DNA binding ability is enabled by NO by bringing their iron-sulphur cluster in their nitrosylated or apo-form [68,124]. *whiB2* and *whiB3* are down regulated in presence of O_2_ while *whiB3*, *whiB6* and *whiB7* are upregulated in the early or late hypoxic response. Of the *whiB* genes, *whiB7* is most upregulated in the macrophage with a 13 fold induction while being 80 fold induced by antibiotics that bind the 30S ribosomal unit [118]. WhiB3 senses NO and O_2_ via its iron-sulphur cluster [73] and regulates genes involved in assimilation of propionate, a by-product of cholesterol degradation, into virulence lipids [125,126,127,128]. Virulence lipids regulated by WhiB3 include sulfolipids, diacyltrehaloses and polyacyltrehaloses, which results in both higher pro- and anti-inflammatory cytokine levels and function as redox sync [126,129]. WhiB3, PhoP and Lsr2 bind to and regulate the *whiB3* operon. MprAB might induce *whiB3* through upregulation of Rv0081, which was predicted to induce the *whiB3* operon [129]. In addition, WhiB3 together with DevSTR regulates expression of *tgs1* which is needed for the production of triacylglycerol, a storage lipid without which *Mtb* cannot resuscitate from dormancy [9,73,130]. WhiB1 is associated with resuscitation as it induces transcription of *whib1*, *rpfA*, *ahpC* and *groEL2* in the absence of NO upon upregulation of WhiB1 by cAMP-CRP [119]. Interestingly, WhiB1 also interacts with GlgB, which is essential for optimal growth of *Mtb*, by reducing intramolecular disulphide bonds [68,119,122].

For a full review of WhiB proteins we refer to the excellent paper by Larsson et al. [118]. For a review of the function of WhiB like proteins and a network view of WhiB1-3 regulated genes and their connection to other virulence factors such as cAMP and CRP we refer to the review by Fei Zheng et al. [68]. An overview of WhiB regulators and the environmental cues they respond to can be found in Figure 2.

**Figure 3:**
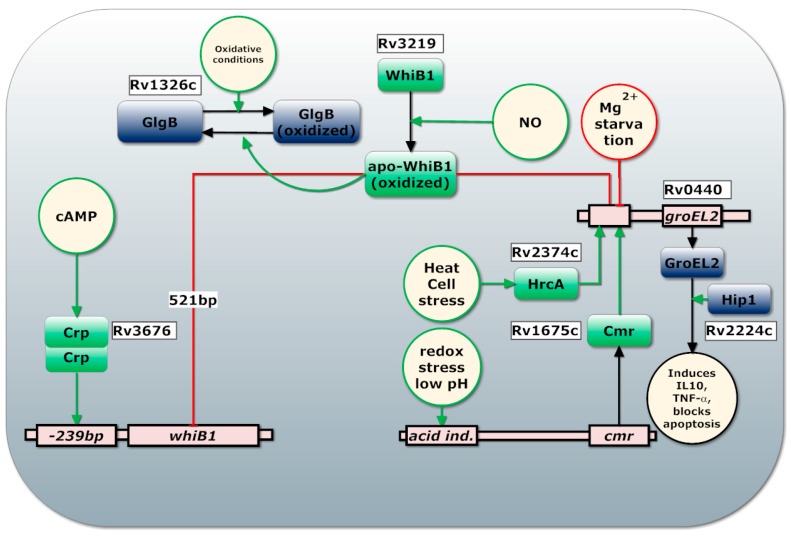
Regulation of GroEL2. Squares represent proteins, circles represent pools of simple chemicals, environmental cues or factors. Green lines indicate induction of transcription while red lines indicate inhibition of transcription. Black lines indicate causal effects.

Two highly regulated virulence systems are EspACD, involved in phagosomal rupture and GroEL2, an abundant chaperonin involved in blocking apoptosis. Regulation of GroEL2 is summarized in Figure 3. GroEL2 is a highly antigenic gene and is associated with increased release of IL-10 and TNF-α which is also associated with cAMP secretion into the cytoplasm of the macrophage [77,79,113,124,131]. GroEL2 forms a dimer and is normally associated to the cell wall. However, Hip1 cleaves cell wall associated GroEL2 to form monomers that are able to cross the phagosome membrane and inhibit apoptosis by interacting with mitochondrial mortalin [132,133]. In this way, Hip1 modulates the macrophage responses by limiting macrophage activation and dampening the activation of TLR2-dependent pro-inflammatory responses [133]. Interestingly, Hip1 has also been reported to function as lipase, making the proteolytic function of Hip1 somewhat disputed [134]. *Mtb* inhibits apoptosis of the macrophage through aggregation of mitochondria around the phagosome and increased activation of mitochondria resulting in limited cytochrome C release, an important inducer of apoptosis [135].

CMR and HrcA positively regulate *groEL2* expression upon acidic and anaerobic stress [124,136]. CRP induces *whiB1* expression in presence of cAMP while WhiB1 represses its own operon as well as *GroEL2* in the presence of NO [124,137]. GroEL2 is therefore only expressed in the presence of cAMP or pH and redox responsive transcription factor CMR or heat stress, while NO is absent (Figure 3). GroEL2 expression is induced 24 h post infection but not at 2 h after infection while other CMR regulated genes, like *Rv1265* and *PE_PGRS6*, are induced at 2 h post-infection [138].

### 3.2. Phagosomal Rupture and Pore Formation

**Figure 4:**
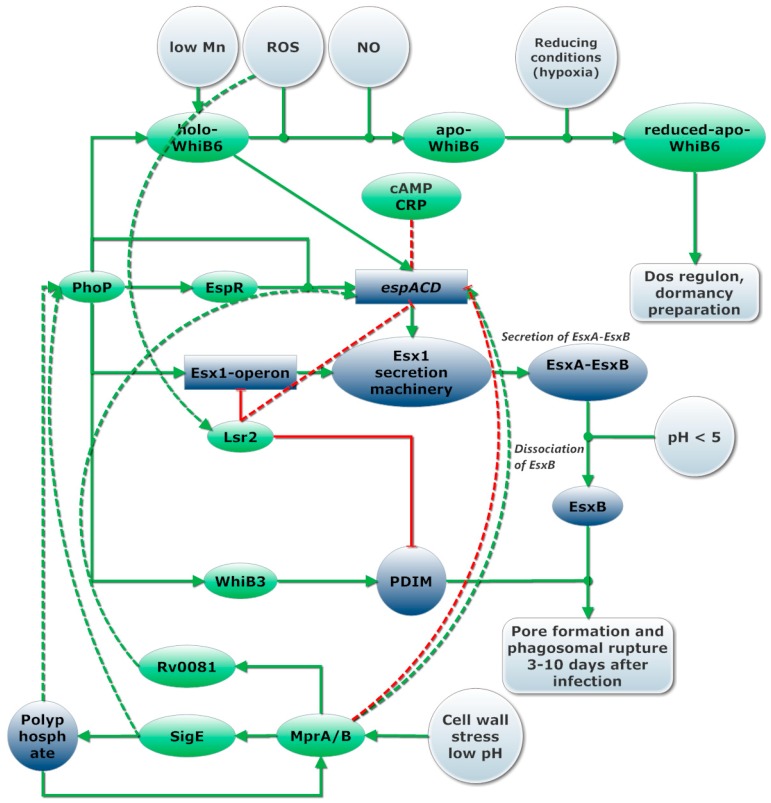
Regulation of pore formation. The circles represent environmental conditions. Arrows indicated regulation (green for induction, red for inhibition of transcription) with dashed lines for uncertain effects. Regulators are depicted in green, proteins and other molecules dark blue while operons are depicted in squares.

The second main virulence strategy deployed by *Mtb* is phagosomal rupture. A model of regulation of pore formation can be found in Figure 4.

ESX-1 and ESX-1 secreted proteins EsxA (ESAT-6) and EsxB (CFP-10) have been implicated in phagosomal rupture of many Mycobateria such as *M. marinum*, *M. kansii* and *Mtb* [139,140,141,142]. The virulence lipid phthiocerol dimycocerosates (PDIM) and EsxA from *Mtb* were shown to interact with the host cell membrane and in concert, induce phagosome membrane damage and rupture in infected macrophages [142,143]. A recent study reported that many claims about pore formation at neutral pH are due to contamination with detergent from the washing step [4]. The same study found membrane-lysing capabilities for EsxA only to occur below pH 5, to be contact dependent and accompanied by gross membrane disruptions rather than discrete pores. For the sake of simplicity, we refer here to the process of cytosolic access as *phagosomal rupture* although more research is needed to find out if cytosolic access is only achieved through lesions or also through formation of pores. Additionally there are reports of *Mtb* and other *Mycobacteria* to escape the phagosome [144]. However, the data generate by electron microscopy—the only direct approach—remains controversial.

The ESX-1 secretion system is involved in secretion of virulence proteins among which those shown to be involved in pore formation and phagosomal rupture EsxA (ESAT-6) and EsxB (CFP-10), secretion associated proteins EspA-D, EspF and secreted immune modulating PE and PPE proteins [96,145,146,147]. Although EsxB is the main pore forming protein, other ESX-1 secreted genes are required for EsxB secretion and proper functioning of the ESX-1 secretion machinery. EspD stabilizes the extracellular levels of EspA and EspC and it is required for EsxA secretion but does not require ESX-1 for its own secretion [148]. Secretion of EspA, EspC, EsxA is codependent on each other, suggesting they might be secreted as a multimeric complex or that they are part of the secretion machinery itself [149,150]. This theory is supported by a study showing that EspA forms dimers by disulphide bond formation after secretion; disruption of this disulphide bond affects cell wall stability as well as the functioning of the whole ESX-1 secretion system [151]. Recently, an EspC-multimeric complex was observed to form filamentous structure that could represent a secretion needle [152]. Inactivation of MyCP1 protease causes hyper-activation of ESX-1 while protease inhibition leads to attenuated virulence during chronic infection [153,154]. A balanced activation and deactivation of ESX-1 through MycP1 proteolysis of EspB is required during chronic infection. MyCP1 and MyCP5 are required for stability of the ESX-1 and ESX-5 secretion complex respectively [155]. Without ESX-1, *Mtb* is unable to disrupt the phagosome membrane and make contact with the cytosol, leading to highly diminished pathogenicity [145].

ESX-1 and secreted factors EsxA and EsxB are regulated by the two-component systems PhoPR, previously mentioned. The importance of PhoP for virulence was confirmed in knockout studies that showed *phoP* knockout mutants to be attenuated in mouse bone marrow derived macrophages, lungs, livers and spleen [156]. A single point mutation in *phoP* in *Mtb* H37Ra decreases the DNA affinity of PhoP and strongly contributes to the reduced virulence of this strain [157]. PhoPR regulated genes are upregulated in acidic and oxidative conditions encountered during the first two days of infection [40]. Recent studies show that PhoP interacts with SigE, which is upregulated in acidic pH and upon cell stress during the first three days of infection [40,158]. Additionally, polyphosphate was needed for normal transcription of *phoP* as well as for transcriptional regulation of *sigE* by *MprAB*, although these results could not be reproduced [159,160]. PhoP/R influences transcription of some 80 (according to some sources up to 150 [161]) genes directly as well as the transcription of a large number of genes indirectly via upregulation of WhiB6, EspR, DevS/R and WhiB3 [60,129].

EspR is a transcriptional regulator upregulated by PhoP. EspR induces transcription of the *espACD* (*Rv3612-16c*) operon which is essential for phagosomal rupture and potential escape from the phago(-lyso)some [148,151,162]. PhoP, therefore, controls, directly (*espB/E-L*) or indirectly (*espA/C/D*), the 13 Esp proteins secreted by ESX-1 [162,163,164]. Recently it was found that holo-WhiB6 increases transcription of its own operon, the ESX-1 regulon and suppressed the DevR regulon, while apo-WhiB6 formed in anaerobic conditions and by prolonged exposure to NO, suppresses the ESX-1 regulon and induces the DevR dormancy regulon [85]. Interestingly, gene expression of EsxB by WhiB6 was highly induced after 30-min of NO exposure, decreased at 60 min and is highly reduced after 3 h of exposure to NO, indicating a short but intense activation of *espACD* by holo-WhiB6. Additionally binding sites for WhiB6 and Rv0081, a transcriptional factor regulated by MprAB, were predicted upstream of *espACD* [84]. These results suggest WhiB6, which is induced by PhoPR and MntR, plays an essential role in the regulation of phagosomal rupture and dormancy.

Induction of transcription of *espACD* by EspR requires the presence of PhoP [162]. In addition, MprAB, Lsr2 and CRP bind to the promotor region of *espACD* operon. Lsr2 represses transcription of both the *espACD* and the ESX-1 operon [84], while CRP binding inhibits expression of *espACD* [165]. Lsr2 binds to AT rich regions in the DNA, mostly virulence genes and is required for adaptation to extreme oxygen conditions [53,54]. We hypothesize it is likely that Lsr2 represses the operon containing ESX-1 genes and *espACD* in oxidative conditions. This could serve to avoid further aggravation of the immune response. MprAB functions as a repressor of the *espACD* operon in cellular stress conditions, however MprA/B is also required for full expression of *espACD.* It is plausible to assume both positive and negative regulation by MprAB occurs based on the presence of multiple binding sites for MprA and two transcriptional start in the *espACD* operon [84].

Like the post-translational activation of GroEL2 by HiP1, membrane lysing capability of EsxA is activated only upon dissociation of EsxA from EsxB in acidic environment (pH 4–5) encountered when the phagosome matures. Acetylation of proteins in *Mtb* is cAMP dependent [141]. Acetylation improves dissociation of EsxA from EsxB at higher pH, a model where acetylation leads to reduced virulence was proposed [166]. Taken together, these studies indicate pore formation is strictly regulated, most likely only occurs when cAMP is depleted (no cAMP-CRP), might be inhibited by sudden changes in oxidative conditions (Lsr2), the phagosome acidifies and become hypoxic (PhoPR) and pore formation is transiently induced by WhiB6 upon NO sensing [85]. MprAB further modifies activation of *espACD*, most likely both positively upon initial cell damage and negatively after prolonged cell stress and accumulation of polyphosphate, as indicated in Figure 4.

It should be mentioned that in addition to their role as regulators, Lsr2, CRP and EspR have also been characterized as nucleoid-associated proteins and as such might serve additional functions such as structuring the organization of the chromosome and, as has been shown for the ESX-1 and espACD operon, protecting DNA region from oxygen radicals [53,165,167].

### 3.3. Dormancy and Modulation of Granuloma Formation

The third virulence strategy deployed by *Mtb* is onset of dormancy. Dormancy is a non-replicating and metabolically near inactive state at which *Mtb* is immune to most drugs and can survive for decades [3,9]. Dormancy occurs upon formation of mostly hypoxic granulomas [168]. Immune modulation that stimulates granuloma formation will therefore be discussed as a part of the dormancy virulence strategy.

When *Mtb* runs out of cAMP to secrete thereby suppressing phagosome lysosome fusion, the macrophages phagosome will fuse with late endosomes and lysosomes. As a result, the phagosome becomes increasingly hostile with lower pH, production of oxygen radicals and NO and fusion with vesicles containing lysozymes. In contrast, conditions encountered in granulomas are slightly more favourable for *Mtb.* Granulomas have reduced capacity to form oxidative radicals [11].

*Mtb* stimulates TNF-α production which leads to granuloma formation among others through secretion of cAMP into the cytosol [70,106,169]. A number of studies indicate that granuloma may be dispensable for preventing bacterial dissemination and may actually contribute to *Mtb* persistence and shield *Mtb* from more successful immune cells [7,10,11]. According to some models, *Mtb* containing granuloma’s contain two types of macrophages: classically activated and alternatively activated [7]. *Mtb* shifts the macrophage population within the granuloma from being classically activated to alternatively activated macrophage which produce more anti-inflammatory cytokines (TGF-β, IL-10) and arginase. These diminish the amount of arginine available to iNOS, which results in reduced NO production [7,11,170]. A balance of pro-inflammatory and anti-inflammatory response via stimulation of TNF-α and IFN-γ production is needed for granuloma formation while IL-10 is the main negative regulator for this response, inhibiting formation of dense and hypoxic mature fibrotic granuloma’s [7,106]. Moreover, parameter sensitivity analysis for a granuloma model, showed IL-10 had the strongest influence on myofibroblast numbers at 300 days post infection and indicated IL-10 to play a major role in preventing differentiation of immune cells needed to develop protective immunity [7,106].

A number of regulators allow *Mtb* to sense and adapt to hypoxia and maturation of the phagosome. The most important of these regulators is the two-component regulator DevRST which regulate genes coding for proteins that help *Mtb* prepare for dormancy and subsequent resuscitation [171,172,173]. A visual representation of DevRST response to environmental cues is present as part of Supplementary File 1. Both DevS and DevT can activate the DevR regulon through phosphorylation of DevR, which autoregulates its own operon through cooperative binding to two binding sites [172,173,174,175]. DevT provides initial activation of the DevR regulon through phosphorylation of DevR and has the strongest sensitivity to CO and a weaker binding to NO and O_2_ compared to DevS. DevS is sufficient for DevR activation after 5 days of infection [176,177]. DevS phosphorylates DevR even in the presence of small concentrations of NO, negatively regulates the DevR regulon through phosphatase activity in the presence of O_2_ while positively regulating the DevR regulon in reducing conditions [176,178,179].

Interestingly, even under non-inducing conditions and as such no phosphorylation of DevR, the DevR regulon is activated upon high enough concentrations of DevR, providing a possible explanation for enduring induction of the DevR regulon which might occur after prolonged autoactivation of its own regulon [175]. Among DevR regulated genes there are a few types of regulation. While some genes are strongly upregulated within a few hours of infection others are only mildly induced after 12–24 h in hypoxic and high NO conditions [174]. DevR and other two-component regulators can fine tune expression of genes through the presence of multiple binding sites and through phosphorylation which stimulates cooperative binding [173].

CO is released by the enzymatic activity of heme oxygenase-1 (HO-1) in lungs infected by *Mtb* [180,181]. CO is an important dormancy inducer. Interestingly, *Mtb* has a unique heme scavenging and degrading systems that does not produce CO allowing *Mtb* to degrade heme without inducing the immune response or its own dormancy regulon.

Interestingly, there is evidence for two DevR regulated proteins to be involved in stabilizing the 30S ribosomal units under hypoxic conditions, while slowing down translation and protein synthesis in the process [168,182]. *Mtb* uses lipids such as cholesterol as primary nutrient in this phase of infection via genes regulated by KstR and IdeR [127,129], while increasing production of triacylglyceride (TAG) via *tgs1* which is under control of DevR and Whib3 [73].

Protein-protein interaction was observed between DevT and NarL, a lone two-component response regulator involved in nitrate and nitrite respiration in *Escherechia coli* [183,184,185]. Although the genes regulated by NarL in *Mtb* are unknown, we argue it is plausible that NarL is involved in regulation of *nirB*, *narU*, *narX*, *narU*, *nuoB* that are currently thought to be part of the DevR regulon.

NO is produced in the maturing phagosome and is an important dormancy cue sensed by DevT and DevS. *Mtb* expresses two truncated heme proteins, GlbN and GlbO, that help it detoxify from nitrate containing oxygen radicals such as NO while residing in the macrophage [186,187,188,189].

Interestingly, *GlbN* is co-transcribed with *lpRl* coding for Lipoprotein LprI, which Acts as a lysozyme inhibitor [190]. The *GlbN-lpR1* Activated isoniazid inhibits truncated haemoglobin N that protects against reactive nitrogen and oxygen species as well as AcpM, which is required for mycolic-acid production [15,191,192,193]. NO was found to help *Mtb* to survive in hypoxic and acidic conditions through anaerobic respiration [185,194]. In addition, nitrate respiration plays an important role in dormancy and protection against hypoxic and acidic stress [194,195].

Although DevRST and WhiB3 are involved in the preparation for dormancy, the enduring hypoxic response measured in a *devR* knockout mutant showed 230 genes to be differentially expressed with roughly half of them upregulated in in the first day of hypoxia and the other half only upregulated at 4 and 7 days of hypoxia [196]. These results indicate many genes involved in the enduring hypoxia response are not regulated by DevR. Resuscitation from dormancy is more elusive and less studied than dormancy. Resuscitation involves ClgR and both SigH and SigE are upregulated upon reaeration [197]. Also cAMP-CRP plays a role in resuscitation as it upregulates *rpfA* one of the five resuscitation promoting factors [137,198,199].

## 4. Success through Tight Regulation of Virulence Strategies

**Figure 5:**
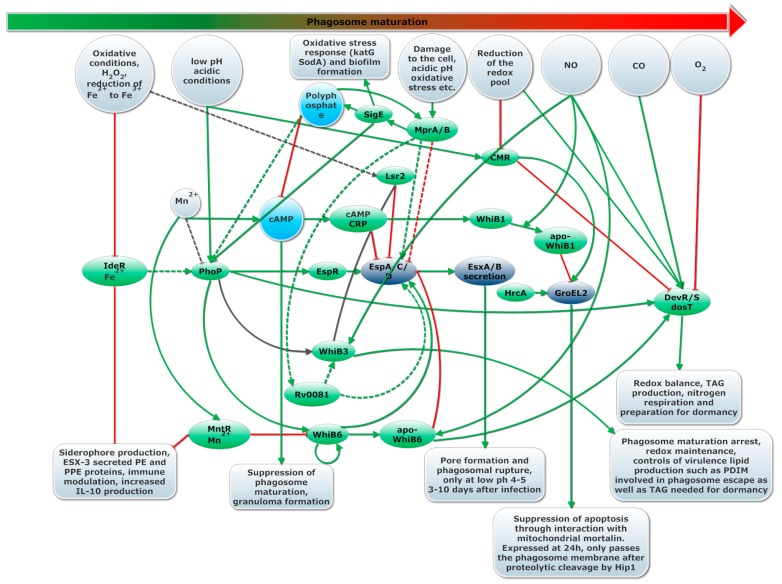
Overview of the regulatory cascade that integrates environmental cues to active the immune modulation, dormancy and phagosomal rupture virulence strategies. Arrows indicated regulation (green for induction, red for inhibition of transcription) with dashed lines for uncertain effects. Regulators are depicted in green, proteins and other molecules dark blue while operons are depicted in squares. The large arrow on the top represents the progression of the immune response.

*Mtb* anticipates changes in the interaction with the host by upregulating both internal and external sensors and regulators involved in sensing progression of the immune response. This allows the bacteria to adjust more quickly to progression of the immune response. External sensors involved in survival in the macrophage consists mostly of two-component regulators [161] (such as DevRST, PhoPR, MprAB, SenX3-RegX3, NarL) while for internal sensors, WhiB family proteins and regulators such as CRP and CMR are used. These sensors and regulators appear interconnected, thus forming a single regulatory cascade that controls the three virulence strategies, as represented in Figure 5. This regulatory cascade integrates many internal (cAMP, Mn, Mg, oxidative conditions and presence of NO) and external environmental cues (phagosome pH or cell wall damage) for fine-tuned regulation of key virulence systems. Examples of such virulence systems downstream this cascade are GroEL2, ESX-1, EsxAB and EspACD. Pore formation by EsxA depends on the regulation of ESX-1 by PhoP, Lsr2 and WhiB6 and on regulation of EspACD by Lsr2, EspR, PhoPR, MprAB, WhiB6 and Rv0081. Post translationally, pore formation by EsxA is regulated by proteolytic activity of MycP1, acetylation of EsxA and dissociation of EsxA-EsxB upon acidification of the phagosome [13,53,54,84,85,139,141,165,166,167]. Similarly, GroEL2 is regulated by CRP, WhiB1, HrCA and Mg^2+^ starvation and post-translationally regulated by proteolytic cleavage by Hip1 [124,132,133,136,137,138].

There is a great amount of overlap in this cascade, so that multiple environmental signals are considered in the regulation of these genes, as indicated in Figure 5. For example, some PhoPR regulated genes are predicted to have cAMP-CRP binding sites [200]. These genes are upregulated upon oxidative stress and low pH but suppressed in the presence of cAMP-CRP, as is the case for *espACD* [201]. Some PhoPR regulated genes are also regulated by DevRST, WhiB3 and by MprAB. An even larger overlap exists in genes regulated by DevRST and MprAB, indicating integration of CO, NO, hypoxia and cell stress in the regulation of these genes [202,203,204]. We argue that based on the overlapping regulation of the three virulence strategies, these strategies extend and overlap each other. The order of activation of these strategies is likely to vary depending on the dynamics between *Mtb* and the host. Timing of specific virulence strategies also vary for different *Mtb* strains [144]. Some strains gain cytosolic access within hours of phagocytosis while others require 3–10 days [13,144].

Pore or lesion formation is linked to immune modulation. Cytosolic access is need for secretion of cAMP and other immune modulating factors, such as GroEL2, into the macrophage cytosol [144]. There are still many unanswered questions regarding the exact role and regulation of GroEL2. Firstly, it is unknown at which conditions proteolysis of GroEL2 by Hip1 (*Rv2224c*) occurs. Secondly, Hip1 was reported to mainly function as lipase in one study [134], further research is needed to confirm whether GroEL2 is a direct substrate of Hip1. Strict regulation of GroEL2 suggests it to have an important role in virulence.

Interestingly, there are many parallels in regulation of virulence systems between *Mtb* and other pathogens. Understanding *Mtb* as one of the most successful intracellular pathogens can therefore provide insight in common strategies deployed by intracellular pathogens. For instance, positive regulation of virulence genes by PhoPR and suppression by cAMP-CRP appears to occur in more pathogens. In *Y. pestis*, PhoP directly binds to and transcriptionally activates *crp* and *cyA* leading to merging of the PhoPQ and CRP-cAMP regulon [205]. Similarly, a major virulence island is positively regulated by PhoP while being suppressed by cAMP-CRP in *S. typhimurium* [206]. In *Mtb*, PhoPR regulates pro-inflammatory virulence genes such as the ESX-1 operon as well as genes involved in protecting against oxidative stress, when cAMP is depleted. cAMP does not only suppress phagosome maturation but also acts as an internal sensor of phagosome maturation, through pH dependent secretion of cAMP.

Some aspects in the regulation of PhoPR and cAMP in *Mtb* require more research. Firstly, the function of multiple IdeR binding sites upstream of the *phoPR* suggests complex regulation of the *phoPR* operon by IdeR and thus by iron bioavailability. Secondly, the exact cue for activation of PhoP remains unknown. Upregulation of *phoPR* in acidic conditions has been observed as well as under Mg^2+^ starvation, however this later observation could not be reproduced [125]. Transcriptional analysis of *Mtb* showed many genes in the PhoPR regulon to be upregulated during the first hours of infection (20 min to 2 h) while the phagosome acidified from pH of 6.5 to pH 5.5 [66]. PhoPR stimulates expression of aprABC, an Mtb specific pH sensing locus involved in the regulation of among others a number of PhoP regulated genes [125]. These results indicated PhoPR directly or indirectly senses pH. Recently, it was discovered that PhoP interacts with acid inducible extracytoplasmic sigma factor SigE, providing a possible explanation for activation of the PhoP regulon at low pH [158]. Extracytoplasmic sigma factors provide a means of regulating gene expression in response to various extracellular changes, hence their name.

Secondly, we argue entrance of *Mtb* in the early phagosome is likely to lead to higher abundance of Mn. Pathogenic *Mycobacteria* species such as *Mtb* and *M. avium*, have high manganese concentrations at 1 and at 24 h after infection compared to non- pathogenic *M. smegmatis* [32]. Mn availability might also be affected by Mramp, a pH dependent Mn H^+^ symporter with maximal activity between pH 5.5 and 6.5 matching the conditions found in the early phagosome. Mn is an important cofactor for cAMP synthesis and it is likely to increase cAMP production in the early phagosome. cAMP-CRP and PhoPR co-regulate virulence genes directly or via regulators such as WhiB6, which is linked to Mn deficiency. Based on the strong affinity of PhoP for Mn we hypothesize Mn might play a role in both cAMP and PhoPR regulation [20,83]. Depletion of Mn and secretion of cAMP might lead to de-repression of cAMP-CRP suppressed genes such as *espACD* as well as activation of these genes through PhoPR.

Thirdly, polyphosphate is needed for optimal PhoP activation [159]. Polyphosphates are potent inhibitors of type III adenylyl cyclases in *M. bovis* which agrees with the opposing roles of cAMP-CRP and PhoPR in respectively inducing genes involved in the anti- and pro-inflammatory response in *Mtb* and other pathogens. Polyphosphate is implicated in the activation of PhoP and is part of one of two positive feedback loops in the regulation of *mprAB* and *sigE* [158,159,160]. Polyphosphates kinase production is conserved in all bacteria and is associated to induction of dormancy and activation of virulence genes in many pathogens [207]. Knockout polyphosphate kinases *ppk1* mutants, have reduced biofilm formation, are more susceptible to drugs and are impaired in growth in guinea pigs [159,208]. Interestingly, SigE is involved in regulation of polyphosphate. MprAB and SigX3-RegX3, induce transcription of *sigE* upon cell wall stress or phosphate starvation, while anti sigma factor RseA binds to and neutralizes SigE in reducing conditions [209,210]. RseA is degraded by ClpC1P2-dependent proteolytic activity depending on its phosphorylation by the eukaryotic-like Ser/Thr protein kinase PknB [210]. SigE, polyphosphate and MprAB are involved in a double positive feedback loops through polyphosphate and ClpC1P2 of which a visual model is provided by Manganelli et al. [210]. Polyphosphate functions as phosphate donor for MprAB under low ATP condition. Additionally, SigE regulates the transcription of the *furA-katG* operon in response to oxidative stress in *Mycobacteria* [67]. SigE knockout strains are strongly attenuated and a recent study shows a *sigE* knockout strain provide an even more effective live vaccine than BCG [211]. Taken together, these studies indicate SigE plays an important role in adapting to low pH, cell wall and oxidative stress through upregulation *furA-katG*, activation of some PhoPR induced genes, MprAB and inhibition of cAMP-CRP through polyphosphate production. The interplay of SigE, polyphosphate and the hypothesized role of Mn in PhoPR and cAMP regulation should be further investigated.

Another aspect we want to address is the link between IdeR, cAMP, cholesterol degradation and phagosomal rupture. IdeR, KstR and KstR2 co-regulate the cholesterol degradation pathway in *M. bovis* [127]. We suggest a similar synergy between IdeR regulation and cholesterol degradation in *Mtb*. Transcription of cholesterol degradation genes in *Mtb* is dependent on the presence of CyA [212]. Regulation of cholesterol degradation by IdeR and cAMP would suggest access to cholesterol is associated to the initial stage of *Mtb* host interaction when the iron pool is oxidized and cAMP is produced to avoid phagosome maturation. Interestingly, EsxA and other pore forming toxins specifically inserts themselves into phosphor lipid (phosphatidylcholine) and cholesterol-containing liposomes [166,213]. Giant foamy macrophages rich in cholesterol are at the centre of *Mtb* containing granuloma’s that turn necrotic [7,11,12,107,213]. Accumulation of cholesterol was shown to be essential for uptake of *Mtb* by the macrophage [214]. Additionally, cholesterol was shown to increase association of TACO, a coat protein that prevents degradation of *Mycobacteria* upon fusion with lysosomes [214]. We argue that accumulation of cholesterol in macrophages not only increases *Mtb* survival in the phagosome by serving as carbon source but also might assists in phagosomal rupture and possibly in escape from the phagosome.

In summary, in this review we provide an overview for understanding divalent metal homeostasis and their role in regulating three essential virulence strategies of *Mtb*: immune modulation, dormancy and phagosomal rupture. Sensors of environmental and internal cues, including divalent metal availability, form a single regulatory cascade that controls these three virulence strategies. The role of polyphosphate, cAMP and manganese in this cascade requires further investigation.

## Figures and Tables

**Table 1:** Suppression of ESX-3 core genes and associated genes by IdeR, Zur and MntR.

Gene	IdeR	Zur	MntR
*esx3-operon* ^1^	−	−	−
*esxG-esxH*	−	−	−
*esxQ*		−	
*esxR-esxS*		−	−
*esxW*			−
*ppe3*		−	−
*ppe4-pe5*	−	−	−
*ppe9*	+		
*pe13*		^2^	−
*ppe19*			−
*ppe20*			−
*ppe37*	−		
*ppe38*		^2^	
*ppe48*		−	
*pe_pgrs61*			−

Plus symbols (+) indicate positive regulation, while minus symbols (−) indicate negative regulation. ^1^ *Rv0282-Rv291*; ^2^ Reported as Zur regulated by Maciag et al. based on direct experimental evidence on two conditions [22]; predicted not to be in the Zur regulon through a large scale analysis of transcriptomics datasets and analysis of binding sites in upstream sequences [99].

## Supplementary Materials

The following are available online at http://www.mdpi.com/1422-0067/19/2/347/s1.

## Abbreviations

AC	Adenylate cyclase
cAMP	Cyclic adenosine monophosphate
CMR	Cyclic-AMP and redox responsive transcription factor
CRP	Cyclic-AMP dependent regulatory protein
DAG	Diacylglycerol
DevRST	DevRST is a two component regulator and sensor, which regulate genes coding for proteins that help *Mtb* prepare for dormancy and subsequent resuscitation
EspR	A virulence associated transcriptional regulator upregulated by PhoP
IdeR	Iron-dependent regulator
Lsr2	A histone like regulator that binds AT-rich regions virulence islands, acting as a global regulator to aid in the adaptation to extremes in oxygen availability
MntR	Manganese-dependent transcriptional repressor
MprAB	A two component sensor and regulator that responds to cell envelop stress
*Mtb*	*Mycobacterium tuberculosis*
PDIM	Phthiocerol dimycocerosates
SigE	Extracytoplasmic alternative Sigma factor E, involved in response to low pH and cell stress
Zur	Zinc uptake regulator
