# Supplementary figures and images for: Regulation of Three Virulence Strategies of Mycobacterium tuberculosis: A Success Story
Source: Int J Mol Sci. 2018 Jan 24;19(2):347. doi: 10.3390/ijms19020347 (PMC5855569; doi:10.3390/ijms19020347)

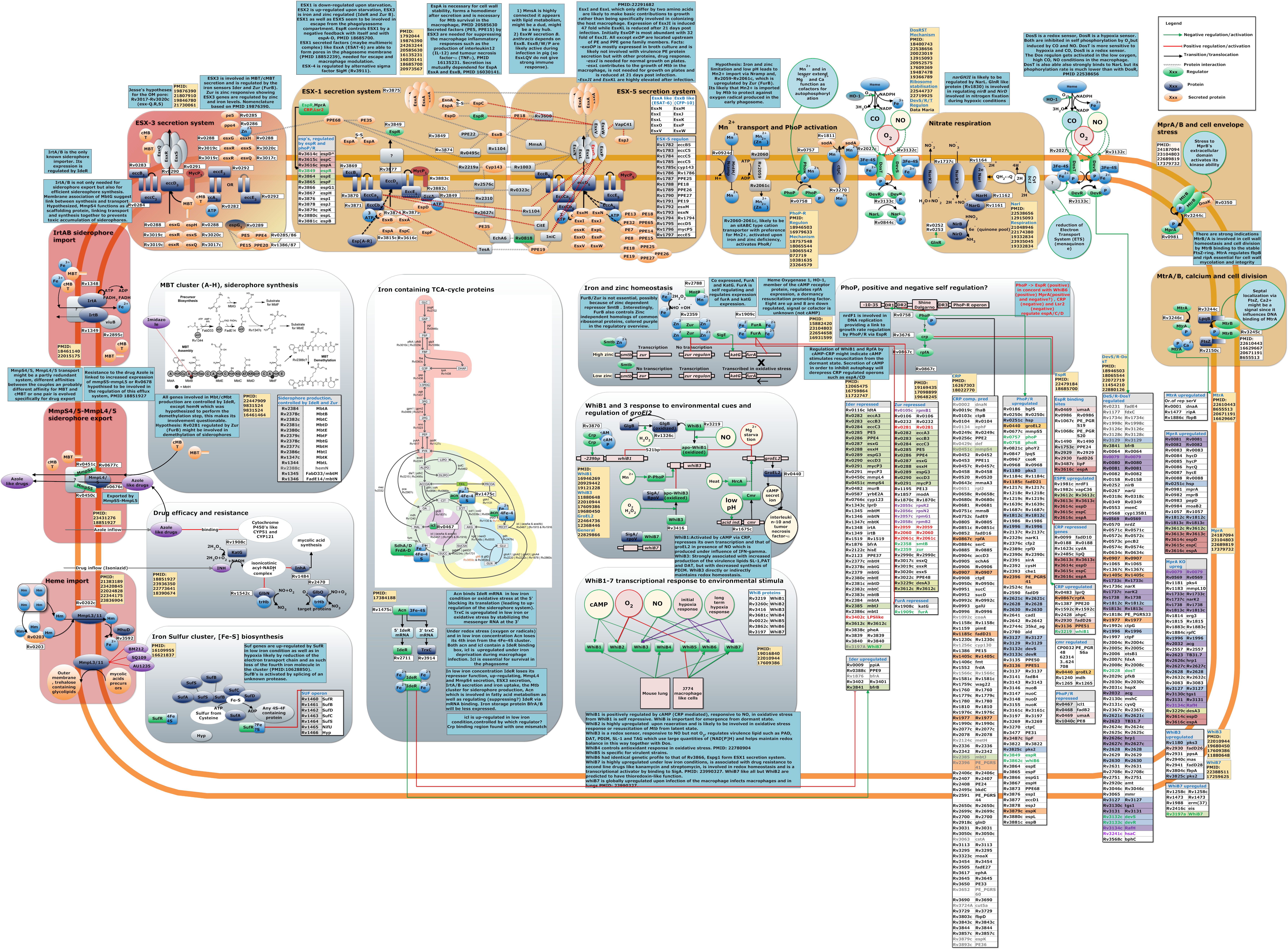

Supplement: Supplementary file 1 [file ijms-19-00347-s001.zip › ijms-262214-supplementary final/Supplementary files/Figure S1.png]
